# Multilocus gene analysis reveals the presence of two phytoplasma groups in *Impatiens balsamina* showing flat stem and phyllody

**DOI:** 10.1007/s13205-021-02666-2

**Published:** 2021-02-11

**Authors:** Priyam Panda, Amrita Nigam, G. P. Rao

**Affiliations:** 1 Discipline of Life Sciences, Indira Gandhi National Open University, New Delhi, 110068 India; 2 Division of Plant Pathology, ICAR-Indian Agricultural Research Institute, New Delhi, 110012 India

**Keywords:** Rose balsam, Aster yellows group, Peanut witches’ broom group, 16S rRNA gene, *secA* gene, *rp* gene, *secY* gene, *tuf* gene, Weeds

## Abstract

Rose balsam (*Impatiens balsamina*) is an important ornamental species grown worldwide for its attractive flowers and also having medicinal properties. Flat stem, little leaf, and phyllody symptoms were observed in *I. balsamina* nurseries in Uttar Pradesh and Tripura states of India during surveys from 2018 to 2020, with an incidence from 6 to 27%. Amplicons of ~ 1.2 kb were amplified in all the tested symptomatic samples of *I. balsamina* using universal phytoplasma primer pairs from different surveyed locations, but not from the asymptomatic plants. Pairwise sequence comparison, phylogeny, and virtual RFLP analysis of 16S rRNA gene sequences identified the phytoplasmas as 16SrI-B subgroup strain from Tripura (Lembucherra) and 16SrII-D subgroup strain from Uttar Pradesh (Gorakhpur and Faizabad). Phytoplasma presence and identity was further confirmed by amplifying *secA*, *rp*, *secY,* and *tuf* genes. This is the first report of 16SrI-B and 16SrII-D phytoplasmas detection in *I. balsamina* in the world.

**Supplementary Information:**

The online version contains supplementary material available at 10.1007/s13205-021-02666-2.

## Introduction

*Impatiens balsamina* L. (Fam: Balsaminaceae), commonly known as rose balsam, is cultivated throughout the world as a seasonal ornamental plant at offices, hotel premises, and social landscapes for its attractive different colour flowers (Staples and Herbst 2005). Different parts of the plant are used as traditional remedies for disease and skin problems (Meenu et al. 2015). Flower crops are affected worldwide by many biotic and abiotic stresses and the phytoplasma associated diseases are the main threats to their commercial cultivations (Bellardi et al. 2018).

Phytoplasmas are cell wall-less prokaryotic microorganisms colonizing plant phloem and insect tissues. The threat of phytoplasma diseases in the world is increasing with a high impact on crop yield, quality and economic losses (Bertaccini et al. 2014; Bertaccini and Lee 2018). In India, six major phytoplasma groups (16SrI, 16SrII, 16SrVI, 16SrIX, 16SrXI, and 16SrXIV) have been identified associated with 34 ornamental plants including rose, chrysanthemum, phlox, petunias, marigold, gladiolus, mussaenda, straw flower, etc. (Madhupriya and Rao 2017; Taloh et al. 2020; Ranebennur et al. 2020; Rao 2021). The occurrence of 16SrI-D and 16SrV-B subgroups of phytoplasma in *I. balsamina* has been reported earlier from China (Chang et al. 2011; Li et al. 2011, 2014).

Although taxonomy based on the highly conserved 16S rRNA gene has been useful for classification purposes, multilocus sequence typing (MLST) provide more detailed differentiation of phytoplasma strains (Hodgetts et al. 2008; Makarova et al. 2012; Martini et al. 2019).

In a recent survey, severe incidence of phytoplasma suspected symptoms of flat stem, little leaf, and phyllody were observed on *I. balsamina* plants in floral nurseries of two states (Uttar Pradesh and Tripura) of India. The present study was undertaken to identify and molecularly characterize the phytoplasmas detected in symptomatic rose balsam plants utilizing multilocus genes analysis and their sequence comparison.

## Materials and methods

Leaves were collected from three symptomatic and two asymptomatic plants of *I. balsamina* showing suspicious phytoplasma symptoms from floral nurseries at campus of Acharya Narendra Deva University of Agriculture and Technology (ANDUAT), Faizabad, Deen Dayal Upadhyaya University (DDUU), Gorakhpur, Uttar Pradesh State and College of Agriculture (CA), Lembucherra, Tripura state during surveys from 2018 to 2020 (Table 1). Weeds showing suspected phytoplasma symptoms in rose balsam nurseries were also collected from the same locations and analyzed to verify phytoplasma presence. The disease incidence was recorded in the field by counting the number of symptomatic rose balsam plants displaying symptoms over asymptomatic plants.

**Table 1:** Survey, symptoms, and identification of phytoplasma stains associated with *Impatiens balsamina* and weeds in different states of India

Host plant	Strains	Location/ State	Symptoms	Survey period	Average disease incidence (%)*	GenBank Acc. No of phytoplasma strains					Group/subgroup identified
						16S rRNA	*secA*	*rp*	*secY*	*tuf*	
*Impatiens balsamina*	IBFS-FAZ1 IBFS-FAZ2	ANDUAT, Faizabad, Uttar Pradesh	Flat stem	October 2018–20	6	MW077123 MW077124	MW071175 MW071176	MW071195 MW071196	MW071185 MW071186	MW071205 MW071206	16SrII-D
*I*. *balsamina*	IBFS-GOK1 IBFS-GOK2	DDUU, Gorakhpur, Uttar Pradesh	Flat stem	October 2018–20	10	MW077125 MW077126	MW071177 MW071178	MW071197 MW071198	MW071187 MW071188	MW071207 MW071208	16SrII-D
*I*. *balsamina*	IBLLP-TRI1 IBLLP-TRI2	CA, Lembucherra, Tripura	Little leaf and phyllody	November 2018–20	27	MW077127 MW077128	MW071183 MW071184	MW071203 MW071204	MW071193 MW071194	MW071213 MW071214	16SrI-B
*Setaria verticillata*	SVLL-FAZ1 SVLL-FAZ2	DDUU, Gorakhpur, Uttar Pradesh	Little leaf	October 2018–20	–	MW077129 MW077130	MW071179 MW071180	MW071199 MW071200	MW071189 MW071190	MW071209 MW071210	16SrII-D
*Cannabis sativa*	CSLY-GOK1 CSLY-GOK2	ANDUAT, Faizabad, Uttar Pradesh	Leaf yellowing	October 2018–20	–	MW077131 MW077132	MW071181 MW071182	MW071201 MW071202	MW071191 MW071192	MW071211 MW071212	16SrII-D

*Average incidence calculated on the basis of visual observation of symptoms in different fields

All samples were stored at 4 °C until processed for DNA extraction. Hundred milligrams of each sample were powdered in liquid nitrogen and total genomic DNA was extracted from leaf midrib and stalk tissue of the plant samples by CTAB method (Ahrens and Seemüller, 1992). The DNA was eluted in 100 μl of elution buffer and kept at − 20 °C to use in PCR assays. PCR analyses were performed in a final reaction volume of 25 μl containing 12.5 µl of OnePCR^™^ 2X PCR Master Mix (GeneDireX, Taiwan), 10.5 µl of nuclease-free water (Sisco Research Laboratories Pvt. Ltd., India), 0.5 µl of each forward/reverse primer 10 pmol/µl (final concentration 0.2 µM), and 1 µl of DNA template (= 50 ng). Direct PCR amplification on 16S rRNA gene was performed using universal phytoplasma primer pairs P1/P7 (Deng and Hiruki 1991; Schneider et al. 1995) followed by nested primer pair R16F2n/R2 (Gundersen and Lee 1996). DNA extracted from the asymptomatic plants were used as negative controls, while DNA extracted from the sesame phyllody phytoplasma maintained on *Catharanthus roseus* in greenhouse (GenBank Acc. No. KC920747) was used as positive control. PCR reactions were carried out in a thermal cycler (Mastercycler, Eppendorf, Hamsburg, Germany) and the cycling protocol used was as reported (Panda et al. 2019).

Because the DNA-based classifications using 16S rRNA gene alone may be insufficient for finer differentiation of phytoplasma strains. Detection on primes amplifying nonribosomal regions, i.e., *rp*, *secY*, and *tuf* genes were also used to confirm and validate phytoplasma presence (Schneider et al. 1997; Lee et al. 2004, 2006, 2010; Martini et al. 2004; Al-Subhi et al. 2018). Moreover, universal phytoplasma specific primers for *secA* gene were also used (Hodgetts et al. 2008; Bekele et al. 2011). The details of different multilocus genes primers along with their amplification products employed in the present study are listed in Supplementary Table 1.

The amplified products of the PCR assays were diluted 1:20 with nuclease free water and 2 µl were used as template in nested PCR assays. Ten microlitres of nested PCR product were subjected to electrophoresis in 1.0% (w/v) agarose gel, stained with GoodView^™^ Nucleic Acid stain (BR Biochem Life Sciences Pvt. Ltd., India) and observed under UV transilluminator. The amplified 16S rDNA fragments were purified using the Wizard^R^ SV Gel and PCR Clean-up System (Promega, Madison, USA). Purified PCR products of 16S rRNA, *secA*, *rp*,* secY,* and *tuf* genes were ligated into pGEM^®^T vector (Promega, Madison, USA) and cloned in competent cells of *Escherichia coli* (DH5-α). The cloned products were outsourced for sequencing using M13Fwd/M13Rev universal primer pair in both directions at Eurofins Genomics India Pvt. Ltd., Bengaluru, Karnataka, India. Qiagen CLC Main workbench was used for sequence data analysis (https://digitalinsights.qiagen.com).

The sequences were assembled using DNA Base V.4 (http://www.dnabaser.com), aligned with phytoplasma ribosomal group/subgroup representatives available in GenBank using ClustalW software, and the consensus sequences were submitted to the GenBank. For ‘*Candidatus* species’ attribution, the 16S rRNA gene sequences were aligned with those of ‘*Ca*. Phytoplasma’ strains, retrieved from NCBI GenBank, and the sequence identity values were determined. A phylogenetic tree was constructed using the neighbor-joining method with MEGA 7.0 (Kumar et al. 2016) using 1,000 bootstrap replications and *Acholeplasma laidlawii* (GenBank Acc. No. AB680603) for 16S rRNA gene and *A*.* oculi* (GenBank Acc. Nos. LK028559:1,196,113–1,198,581, LK028559:1,475,939–1,476,271, LK028559:1,468,847–1,470,145, LK028559:1,398,525–1,399,706) for *secA*, *rp*, *secY,* and *tuf* genes, respectively to root the trees.

About ~ 1.25 kb of phytoplasma sequences corresponding to the R16F2n/R2 fragments of *I. balsamina* and weeds phytoplasma strains were subjected to in silico RFLP comparison analysis using the *i*PhyClassifier online tool and similarity coefficient value was calculated (Zhao et al. 2009).

## Results

**Fig. 1:**
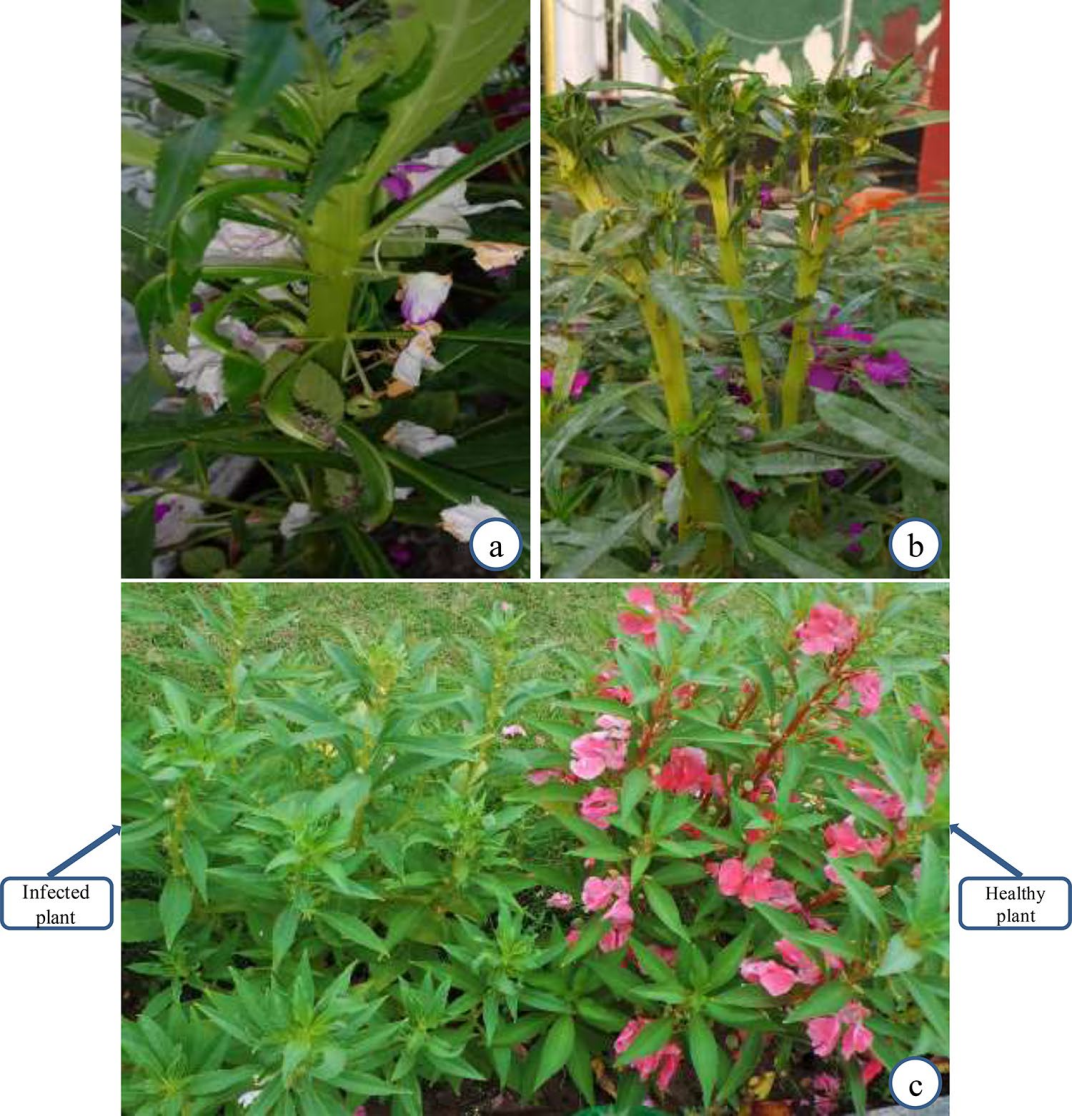
*Impatiens balsamina* plants showing phytoplasma symptoms **a** flat stem at ANDUAT, Faizabad; **b** flat stem at DDUU, Gorakhpur, U.P.; **c** little leaf and phyllody at CA, Lembucherra, Tripura

A typical flat stem symptom was recorded on *I. balsamina* plants grown at floral nurseries of ANDUAT, Faizabad (IBFS-FAZ) and DDUU, Gorakhpur (IBFS-GOK), Uttar Pradesh (Fig. 1a, b) with disease incidence of 6–10% (Table 1). Moreover, little leaf and phyllody symptoms were noticed on *I. balsamina* plants at college campus of  Lembucherra, Tripura (IBLLP-TRI) (Fig. 1c) with disease incidence up to 27% (Table 1). *Setaria verticillata* grown as weed in rose balsam nursery at DDUU, Gorakhpur was recorded with little leaf symptoms. Leaf yellowing symptoms were also recorded in *Cannabis sativa* weed grown nearby balsam nursery at ANDUAT, Faizabad.

An amplification of ~ 1.8 kb and  ~ 1.25 kb was consistently obtained from all the nine symptomatic *I. balsamina* samples collected from Faizabad, Gorakhpur, and Lembucherra locations along with the positive controls in nested PCR assays using primer pairs P1/P7 followed by R16F2n/R2, respectively, but not in any of the asymptomatic rose balsam samples from the three places (data not shown).

Similar amplifications of phytoplasma DNA were achieved from both the collected symptomatic weed species (*S*. *verticillata* and *C*. *sativa*) (two samples each) from Gorakhpur and Faizabad, Uttar Pradesh. The sequences of the two-rose balsam phytoplasma strains from all the three locations and the two weeds were analyzed, edited, and deposited in GenBank (Table 1).

Pairwise comparison of 16S rRNA gene sequences corresponding to R16F2n/R2 fragment of rose balsam phytoplasma with the corresponding regions of different phytoplasma strains retrieved from NCBI database belongs to diverse ribosomal groups indicated that the IBLLP-TRI phytoplasma stains (GenBank Acc. Nos. MW077127-28) shared maximum nucleotide identity ranging between 99.60% and 100% with earlier reported aster yellows (16SrI) group related phytoplasma strains belonging to *Zinnia elegans* yellows (GenBank Acc. No. MN379838), sesame phyllody (GenBank Acc. No. KC920749) and periwinkle virescence (GenBank Acc. No. FN257484). However, IBFS-FAZ and IBFS-GOK phytoplasma strains (GenBank Acc. Nos. MW077123-26) shared 100% sequence identity among themselves and with earlier reported peanut witches’ broom (16SrII) group related phytoplasma strains reported earlier with faba bean phyllody (GenBank Acc. No. MK453522), chickpea phyllody (GenBank Acc. No. MN551487) and papaya crinkle yellow (GenBank Acc. No. Y10096). Pairwise comparison of 16S rRNA gene sequences of phytoplasma strains detected in the two weed samples viz., *S. verticillata* little leaf (SVLL-GOK) (GenBank Acc. Nos. MW077129-30) and *C*. *sativa* leaf yellowing (CSLY-FAZ) (GenBank Acc. Nos. MW077131-32) shared 100% sequence identity with peanut witches’ broom (16SrII) group related phytoplasma strains.

**Fig. 2:**
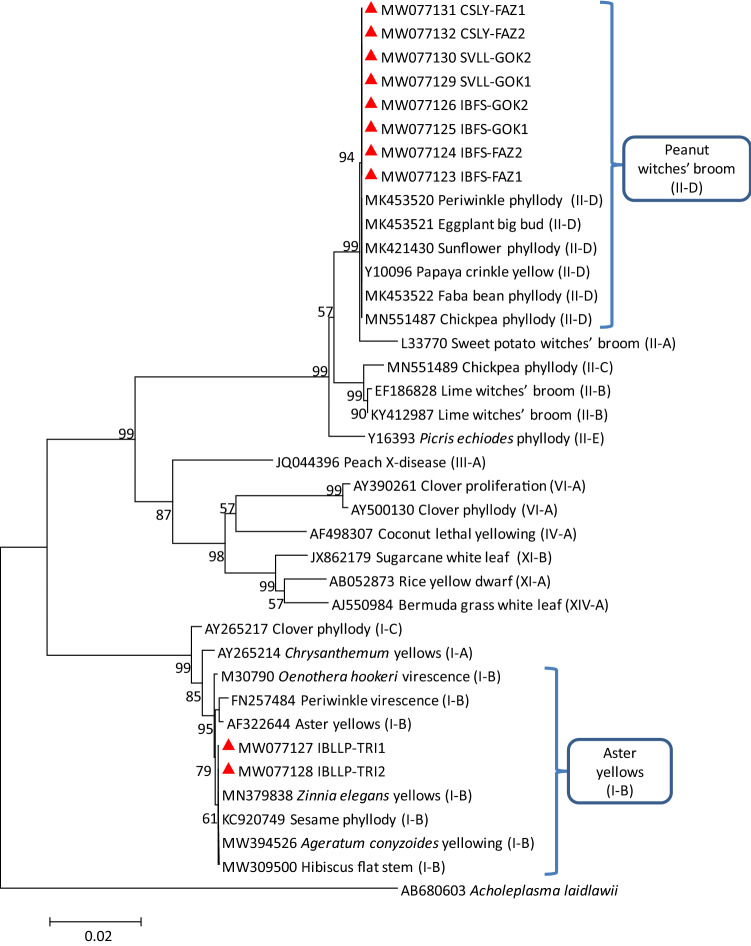
Phylogenetic tree constructed by neighbor-joining method of the partial 16S rRNA gene sequences from 16SrI and 16SrII group of phytoplasma strains, *Impatiens balsamina* little leaf and phyllody (IBLLP-TRI), *Impatiens balsamina* flat stem (IBFS-FAZ and IBFS-GOK), *Setaria verticillata* little leaf (SVLL-GOK), and *Cannabis sativa* leaf yellowing (CSLY-FAZ) (red triangles). Bootstrapping was conducted 1000 times and *Acholeplasma laidlawii* was included as outgroup. A number on branches indicate confidence values associated with the bootstrap analysis

The pairwise comparison results were well supported by the corresponding phylogenetic sequence analysis of 16S rRNA gene in which IBLLP-TRI strains clustered with 16SrI group related strains and IBFS-FAZ, IBFS-GOK, SVLL-GOK, and CSLY-FAZ strains clustered with 16SrII group related strains (Fig. 2).

The PCR amplicons of ~ 600 bp for *secA*, ~ 1200 bp for *rp*, ~ 1400 bp for *secY,* and ~ 940 bp for *tuf* genes were obtained in symptomatic rose balsam plants samples from Lembucherra, Tripura by using 16SrI group specific primers (Supplementary Table 1). Whereas amplicons of ~ 600 bp for *secA*, ~ 1300 bp for *rp*,  ~ 1700 bp for *secY,* and ~ 1094 bp for *tuf* were consistently obtained with rose balsam and weeds symptomatic samples from Faizabad and Gorakhpur using 16SrII group specific primers (Supplementary Table 1). The nested multilocus genes amplified PCR products were sequenced, analyzed, and partial *secA*, *rp*, *secY,* and *tuf* gene sequences were deposited in the GenBank database (Table 1).

Pairwise sequence comparison of *secA* gene of IBLLP-TRI phytoplasma stains (Table 1) was compared with different phytoplasma strains retrieved from NCBI database and showed maximum nucleotide identity of 99.4–100% with several phytoplasma classified in 16SrI group infecting sesame phyllody, periwinkle virescence, oil palm stunt, and sugarcane yellows, while IBFS-FAZ, IBFS-GOK, and weeds phytoplasma strains (Table 1) shared 100% sequence identity with each other and 99.37–100% identity with other previously identified phytoplasma classified in 16SrII group infecting chickpea phyllody, mango malformation, and guava leaf yellowing. Similarly, the *rp*, *secY,* and *tuf* gene sequences of IBLLP-TRI phytoplasma stains (Table 1) showed maximum identities ranging between 99.3% and 100% with previously reported phytoplasma strains classified in 16SrI group, while IBFS-FAZ, IBFS-GOK, and weeds phytoplasma strains (Table 1) shared sequence identity ranging between 99.82% and 100% with previously identified phytoplasma strains enclosed in 16SrII group.

**Fig. 3:**
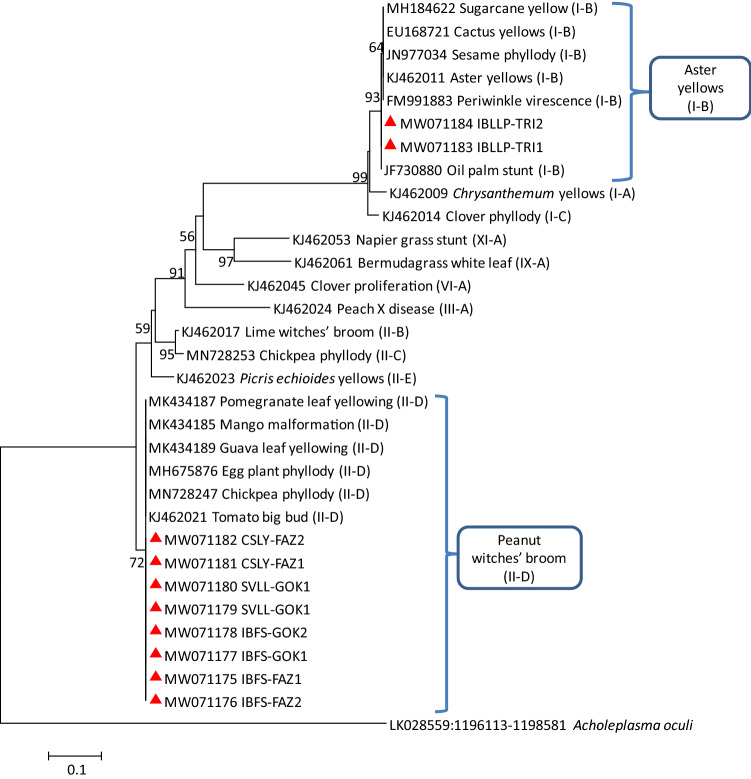
Phylogenetic tree constructed by neighbor-joining method of the partial *secA* gene sequences from I and II group of phytoplasma strains, *Impatiens balsamina* little leaf and phyllody (IBLLP-TRI), *Impatiens balsamina* flat stem (IBFS-FAZ and IBFS-GOK), *Setaria verticillata* little leaf (SVLL-GOK) and *Cannabis sativa* leaf yellowing (CSLY-FAZ) (red triangles). Accession numbers are specified in the tree and *Acholeplasma oculi* was used as outgroup. A number on branches are bootstraps values obtained for 1000 replicates

**Fig. 4:**
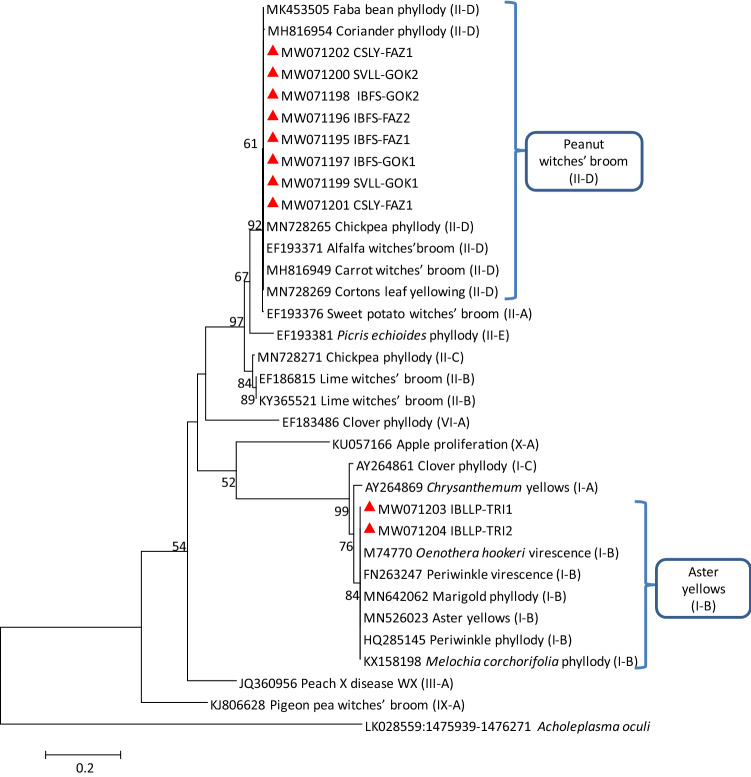
Phylogenetic tree constructed by neighbor-joining method of the *rp* gene sequences from I and II group of phytoplasma strains, *Impatiens balsamina* little leaf and phyllody (IBLLP-TRI), *Impatiens balsamina* flat stem (IBFS-FAZ and IBFS-GOK), *Setaria verticillata* little leaf (SVLL-GOK), and *Cannabis sativa* leaf yellowing (CSLY-FAZ) (red triangles). Accession numbers are specified in the tree and *Acholeplasma oculi* was used as outgroup. A number on branches are bootstraps values obtained for 1000 replicates

**Fig. 5:**
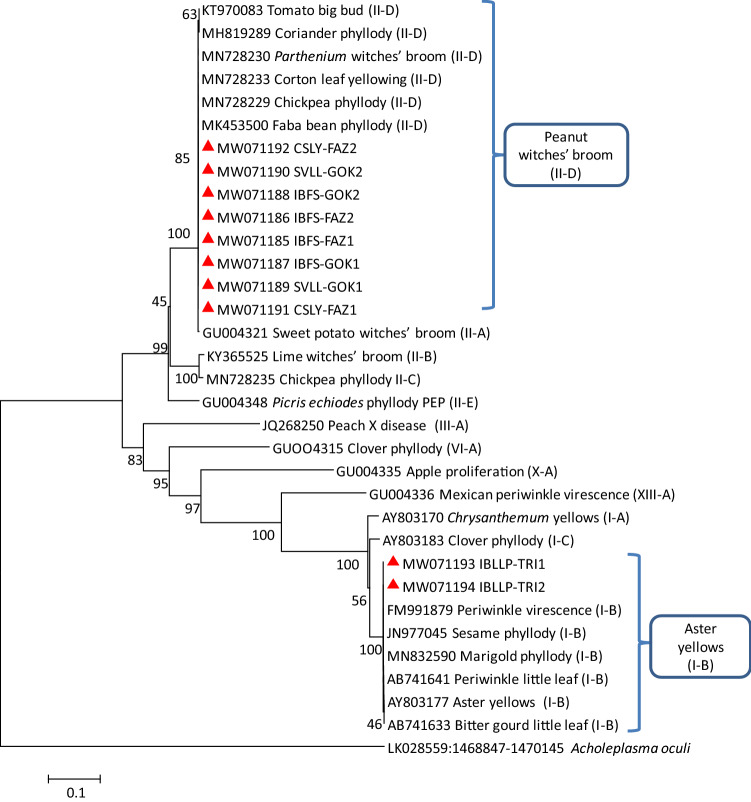
Phylogenetic tree constructed by neighbor-joining method of the *secY* gene sequences from I and II group of phytoplasma strains, *Impatiens balsamina* little leaf and phyllody (IBLLP-TRI), *Impatiens balsamina* flat stem (IBFS-FAZ and IBFS-GOK), *Setaria verticillata* little leaf (SVLL-GOK), and *Cannabis sativa* leaf yellowing (CSLY-FAZ) (red triangles). Accession numbers are specified in the tree, and *Acholeplasma oculi* was used as outgroup. A number on branches are bootstraps values obtained for 1000 replicates

**Fig. 6:**
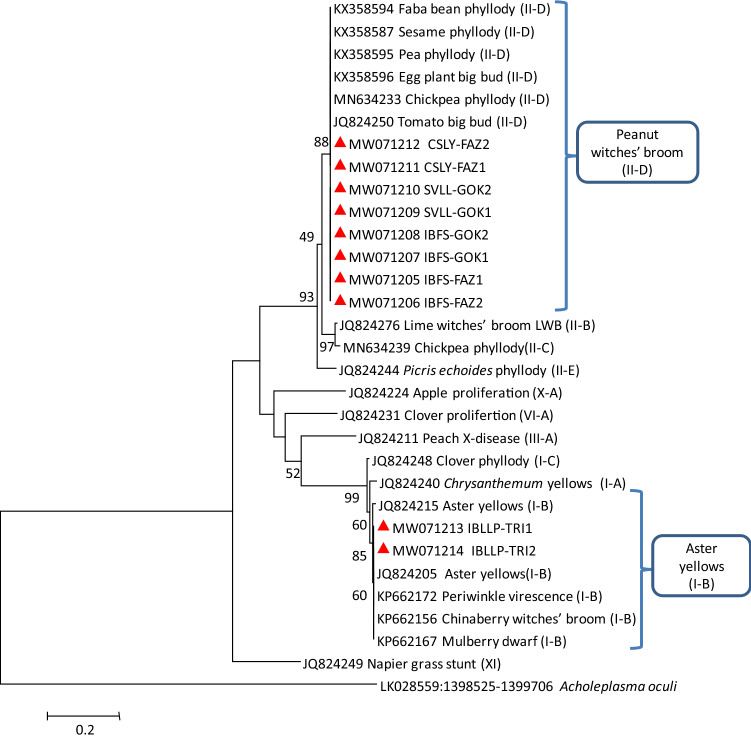
Phylogenetic tree constructed by neighbor-joining method of the partial *tuf* gene sequences from I and II group of phytoplasma strains, *Impatiens balsamina* little leaf and phyllody (IBLLP-TRI), *Impatiens balsamina* flat stem (IBFS-FAZ and IBFS-GOK), *Setaria verticillata* little leaf (SVLL-GOK), and *Cannabis sativa* leaf yellowing (CSLY-FAZ) (red triangles). Accession numbers are specified in the tree and *Acholeplasma oculi* was used as outgroup. A number on branches are bootstraps values obtained for 1000 replicates

These results were also supported by the corresponding phylogenetic sequence analysis of *secA*, *rp*, *secY*, and *tuf* genes in which IBLLP-TRI strain was clustered with 16SrI group related strains and IBFS-FAZ, IBFS-GOK, SVLL-GOK, and CSLY-FAZ strains were clustered with 16SrII group related strains (Figs. 3, 4, 5, 6).

**Fig. 7:**
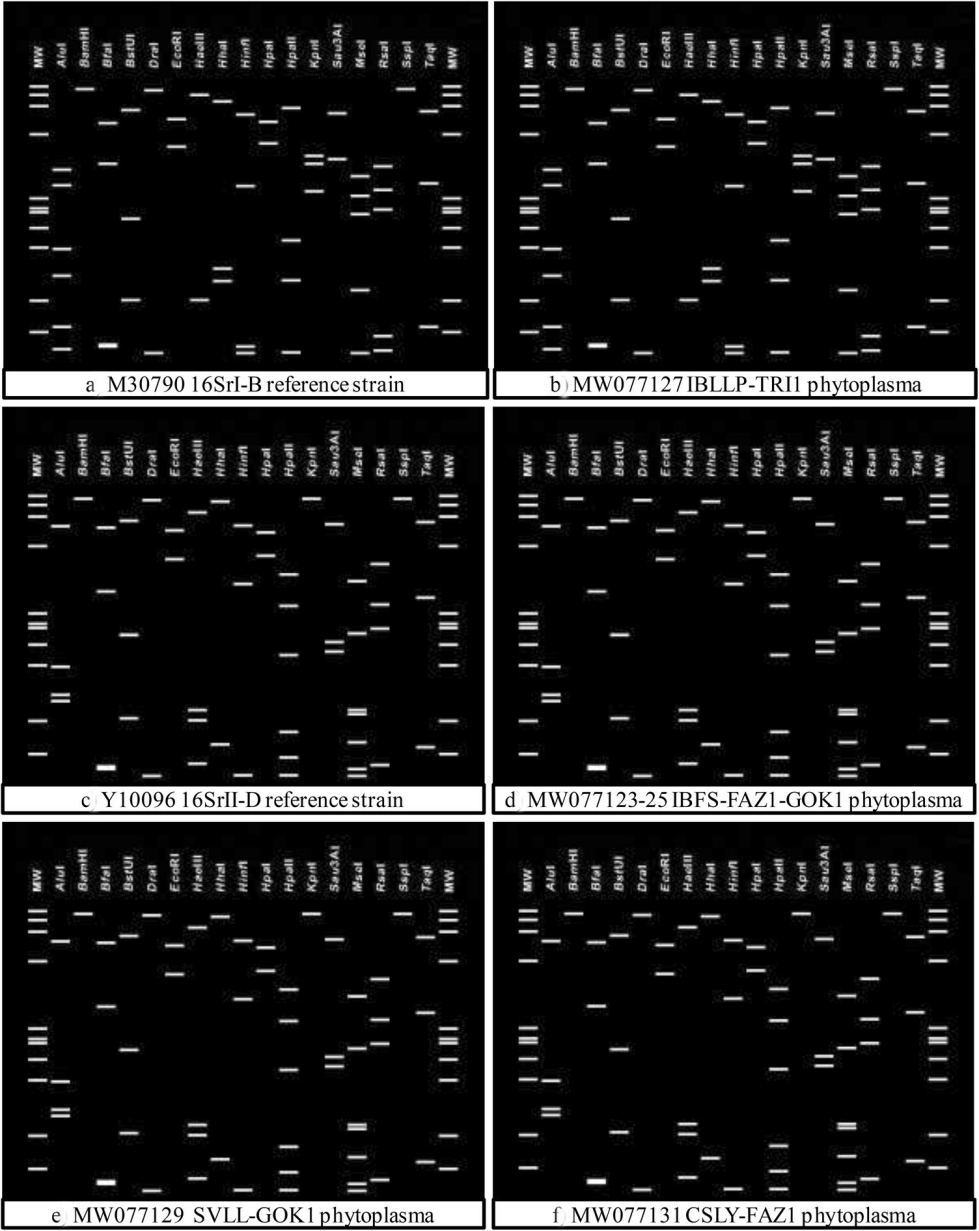
Comparison of virtual RFLP patterns derived from in silico digestion of ~ 1.25 kb 16S rRNA gene sequences of reference phytoplasmas subgroup with 17 different restriction endonucleases using *i*phyclassifier programme, **a** 16SrI-B reference strain (GenBank Acc. No. M30790), **b** *Impatiens balsamina* little leaf and phyllody (IBLLP-TRI1), **c** 16SrII-D reference strain (GenBank Acc. No. Y10096), **d** *Impatiens balsamina* flat stem (IBFS-FAZ1 and IBFS-GOK1), **e** *Setaria verticillata* little leaf strain (SVLL-GOK1), and **f** *Cannabis sativa* leaf yellowing (CSLY-FAZ1)

Virtual RFLP analysis results derived from in silico digestions of R16F2n/R2 region of 16S rRNA gene using 17 restriction endonucleases enzymes (*Alu*I, *Bam*HI, *Bfa*I, *Bst*UI, *Dra*I, *Eco*RI, *Hae*III, *Hha*I, *Hin*fI, *Hpa*I, *Hpa*II, *Kpn*I, *Mbo*I, *Mse*I, *Rsa*I, *Ssp*I, and *Taq*I) indicated that IBLLP phytoplasma stains from Tripura (GenBank Acc. Nos. MW077127-28) were produced similar virtual RFLP profile identical to reference strain for 16SrI-B subgroup (GenBank Acc. No. AP006628) (Fig. 7a, b) with a similarity coefficient of 1.0. However, IBFS (GenBank Acc. Nos. MW077123-26) and two weeds (GenBank Acc. Nos. MW077129-32) phytoplasma strains from Faizabad and Gorakhpur generated restriction patterns identical to that of reference phytoplasma strain, 16SrII-D subgroup (GenBank Acc. No. Y10096) (Fig. 7c–f) with a similarity coefficient of 1.0. On the basis of similar restriction profiles, the rose balsam and weeds phytoplasma isolates in this study were classified under16SrI-B and 16SrII-D subgroups related phytoplasmas strains.

## Discussion

There are thousands of 16S rRNA gene sequences of phytoplasma deposited in the public databases as well as sequences to other conserved genomic regions used as supplementary tools for finer taxonomic differentiation (Duduk and Bertaccini 2011). The *rp*, *tuf*, *secA,* and *secY* genes are reported to show more variation than the 16S rRNA gene and are hence useful for the finer genetic diversity among the phytoplasma strains (Bertaccini and Lee 2018; Martini et al. 2019). Keeping this in mind, in the present study, four genes (*secA*, *rp*, *secY,* and *tuf*) other than 16S rRNA were used to confirm the presence of two subgroups of phytoplasmas (16SrI-B and 16SrII-D) in symptomatic rose balsam plants and two weed samples collected from three different locations in India. The results further confirmed the validity and utility of these four genes as additional molecular markers for characterization of phytoplasma strains belonging to 16SrI-B and 16SrII-D subgroups. Multilocus genes have been successfully used earlier for finer differentiation of closely related subgroup phytoplasmas strains (Martini et al. 2007; Bohunická et al. 2018; Siampour et al. 2019). However, in the present study, all the four genes used provided similar results of grouping and subgrouping classification of rose balsam phytoplasma strains indicating that the rose balsam phytoplasma strains identified from two states of India do not have significant genetic diversity among themselves. Earlier, only two reports of occurrence of 16SrI-D and 16SrV-B subgroups of phytoplasmas are available reporting the association of phyllody and virescence disease of rose balsam from Yangling and Shandong provinces of China (Chang et al. 2011; Li et al. 2011, 2014). The present findings expand the current knowledge regarding distribution of aster yellows and peanut witches’ broom related phytoplasma strains in a new agro ecosystem and report rose balsam as a new host of 16SrI-B and 16SrII-D subgroup of phytoplasma in the world.

The 16SrI-B and 16SrII-D subgroup, reported in rose balsam and weeds have already been reported as a major widespread phytoplasma strains infecting several important crops viz. vegetables, fruits, ornamentals, legumes, and spices in India (Kumar et al. 2017; Mitra et al. 2019; Rao et al. 2019; Panda et al. 2020; Rihne et al. 2020; Rao 2021). This study also suggested the role of *S*. *verticillata* and *C*. *sativa* weeds growing around the *I. balsamina* nurseries in Gorakhpur and Faizabad locations which may act as a natural host reservoir for the transmission of 16SrII-D phytoplasmas. Since the rose balsam is an important seasonal ornamental crop being grown in all parts of the country, the reported phytoplasma weed hosts may facilitate transmission of phytoplasma strains associated with rose balsam to other crops with help of leafhoppers in the country. Different species of leafhoppers (*Hishimonus phycitis*, * Orosius albicinctus*, *Amarasca bigutella, Empoasca motti*) are already identified as putative or natural vectors of phytoplasma strains belonging to 16SrI-B and II-D subgroups in India (Rao 2021) and may play significant role in transmission of rose balsam phytoplasma strains to other important agricultural crops.

The scenario of wider natural spread of phytoplasma strains 16SrI-B and 16SrII-D infecting several crop species in India may pose a serious threat for other agriculturally important plants grown nearby rose balsam flower nurseries and thus have a great epidemiological significance. Further investigations are required to know the major insect vectors and weed species involved in the natural propagation of these phytoplasma strains to develop suitable control measures.

## Supplementary Information

Below is the link to the electronic supplementary material.

Supplementary file1 (DOCX 18 KB)
