# Supplementary material for: Multilocus gene analysis reveals the presence of two phytoplasma groups in Impatiens balsamina showing flat stem and phyllody
Source: 3 Biotech. 2021 Feb 11;11(3):122. doi: 10.1007/s13205-021-02666-2 (PMC7878611; doi:10.1007/s13205-021-02666-2)
Supplement: Supplementary file 1 — Supplementary file1 (DOCX 18 KB) [file 13205_2021_2666_MOESM1_ESM.docx]

Supplementary Table 1 Universal and group specifics primers used for PCR amplification and sequencing of the *secA*, *rp*, *secY* and *tuf* genes of group I and II of *Impatiens balsamina* and weeds phytoplasmas

| Primer set | Sequence (5’ to 3’) | 16Sr group phytoplasma specificity | Location | PCR product size (bp) | Reaction | Reference |
| --- | --- | --- | --- | --- | --- | --- |
| SecAfor1/  SecArev3 | GARATGAAAACTGGRGAAGG/  GTTTTRGCAGTTCCTGTCATNCC | Universal | *secA* gene | 840 | Direct PCR | Hodgetts et al. 2008 |
| SecAfo5r/  SecARev2 | CTGATAGAGAAGCTAATGG/ CCNTCRCTAAATTGNCGTCC | Universal | *secA* gene | 600 | Nested PCR | Bekele et al. 2011 |
| rpF1/  rpR1 | GGACATAAGTTAGGTGAATTT/  ACGATATTTAGTTCTTTTTGG | Group I, III, IV, V, VII,  VIII, IX, XIII | *rpsS*/ *rplP* | 1,245–1,389 | Direct PCR | Lee et al. 2004 |
| rp(I)F1A/ rp(I)R1A | TTTTCCCCTACACGTACTTA/  GTTCTTTTTGGCATTAACAT | Group I | *rpsS,*  *rpsC* / *rplP* | 1200 | Nested PCR | Lee et al. 2004 |
| AYsecYF1/  AYsecYR1 | CAGCCATTTTAGCAGTTGGTGG  CAGAAGCTTGAGTGCCTTTACC | Group I | *secY* gene | 1400 | Direct PCR | Lee et al. 2006 |
| fTuf1/  rTuf1 | CACATTGACCACGGTAAAAC/  CCACCTTCACGAATAGAGAAC | Group I | *tuf* gene | 1038 | Direct PCR | Schneider et al. 1997 |
| fTufAY/  rTufAY | GCTAAAAGTAGAGCTTATGA/  CGTTGTCACCTGGCATTACC | Group I | *tuf* gene | 940 | Nested PCR | Schneider et al. 1997 |
| rp(II)F/  rp(I)R1A | ACTTATTCTCGTGATACTAG/  GTTCTTTTTGGCATTAACAT | Group II | *rpsS* *rpsC* / *rplP* | 1390 | Direct PCR | Martini, 2004 |
| rp(II)F2/  rp(I)R1A | ATGGTAGGTTATAAATTAGG/  GTTCTTTTTGGCATTAACAT | Group II | *rpsS* *rpsC* / *rplP* | 1290 | Semi -nested PCR | Martini, 2004 |
| SecYF1(II)/  SecYR1(II) | CGCGTATAGGTTTTGAAGGTG/  CCTGCCATTTTCATTATAGCG | Group II | *secY* gene | 2200 | Direct PCR | Lee et al. 2010 |
| SecYF2 (II)/  SecYR1(II) | TGAAGGTGGTCAAACTCCT/  CCTGCCATTTTCATTATAGCG | Group II | *secY* gene | 1700 | Semi-nested PCR | Lee et al. 2010 |
| TUF-II-F1/ TUF-II-R1 | GCTTTTGTTCCTTTAGCAGAA/ AGACTATACACTAGTCTTCTT | Group II | *tuf* gene | 1490 | Direct PCR | Al-Subhi et al. 2018 |
| TUF-II-F2/ TUF-II-R1 | CGCAAAGATATTAAAACTTTAG/ AGACTATACACTAGTCTTCTT | Group II | *tuf* gene | 1094 | Semi-nested PCR | Al-Subhi et al. 2018 |
